# A comprehensive annotation for the root-knot nematode *Meloidogyne incognita* proteome data

**DOI:** 10.1016/j.dib.2018.05.131

**Published:** 2018-05-26

**Authors:** Vishal Singh Somvanshi, Olivia Ghosh, Roli Budhwar, Bhoomika Dubay, Rohit Nandan Shukla, Uma Rao

**Affiliations:** a Division of Nematology, LBS Center, ICAR-Indian Agricultural Research Institute, PUSA Campus, New Delhi 110012, India; b Bionivid Technology Private Limited, 209, 4th Cross, Kasturi Nagar, Bangalore 560043, India

## Abstract

Root-knot nematodes are devastating pathogens of crop plants. The draft genome of southern root-knot nematode *Meloidogyne incognita* was published in 2008 and additional genome and transcriptome data became available later on. However, lack of a publically available annotation for *M. incognita* genome and transcriptome(s) limits the use of this data for functional and comparative genomics by the interested researchers. Here we present a comprehensive annotation for the *M. incognita* proteome data available at INRA Meloidogyne Genomic Resources page (https://meloidogyne.inra.fr/Downloads/Meloidogyne-incognita-V2-2017) and European Nucleotide Archive (ENA) (accession number: ERP009887) using a multi-pronged approach.

## Specifications Table

**Table:** 

Subject area	*Agricultural Sciences, Biology*
More specific subject area	*Nematode Genomics*
Type of data	*Table, text file, figure, MS Excel sheet*
How data was acquired	*The protein sequence data was obtained from INRA Meloidogyne Genomic Resources page* (https://meloidogyne.inra.fr/Downloads/Meloidogyne-incognita-V2-2017). *The corresponding nucleotide sequences are available at European Nucleotide Archive (ENA) accession number*ERP009887*and can be accessed at*https://www.ebi.ac.uk/ena/data/search?query=ERP009887
Data format	*Analyzed*
Experimental factors	*Annotation of M. incognita proteome by using multiple approaches*
Experimental features	*Secondary analysis*
Data source location	*INRA Meloidogyne Genomic Resources* (https://meloidogyne.inra.fr/Downloads/Meloidogyne-incognita-V2-2017)
Data accessibility	*The data arising out of our analysis is attached with this article as tables and figures.*

## Value of the data

• Lack of a publically available annotation for *Meloidogyne incognita* genomic and transcriptomic data is a major limitation for its direct use by the broader scientific community.
• A comprehensive annotation for the *M. incognita* proteome is presented using a multi-pronged approach. As compared to the 67.7% of the total proteins annotated by the standard approach using RefSeq database, the multi-pronged approach resulted in annotation of 73% of the proteome.
• The annotation of *M. incognita* proteome data can be helpful for a large number of researchers who are using RNA-seq data for understanding the biology of *M. incognita* for applied purposes. The availability and access of this annotation would help the researchers globally in a manner that they need not assemble their RNA-Seq data to construct transcriptome for various experiments and then annotate it. Instead, the researchers can simply map their RNA-Seq data to the available cDNA using recent tools such as Kallisto, Salmon, Sailfish etc., and use the provided annotation to interpret their experimental findings quickly.
• Present annotation would save significant time and computing resources required for the assembly and annotation, and allow the researchers to focus on answering the biological questions faster. This would be highly beneficial for development of novel strategies to combat this global pest menace.

## 1 Data

**Fig. 1:**
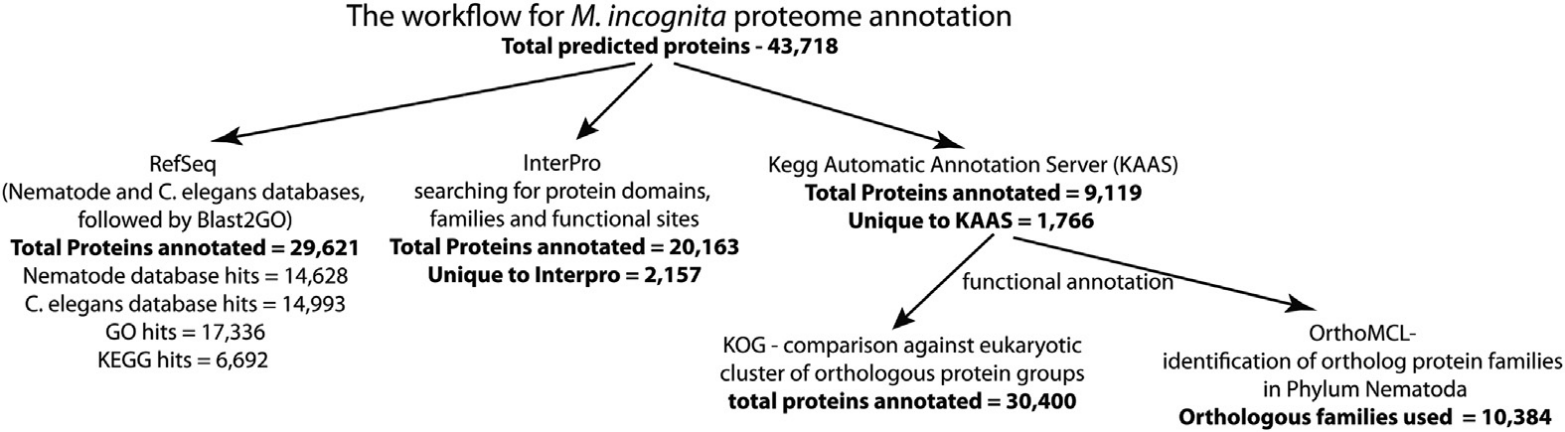
A summary of the approaches used to annotate *Meloidogyne incognita* proteome. The number of proteins annotated by each method are also shown.

**Fig. 2:**
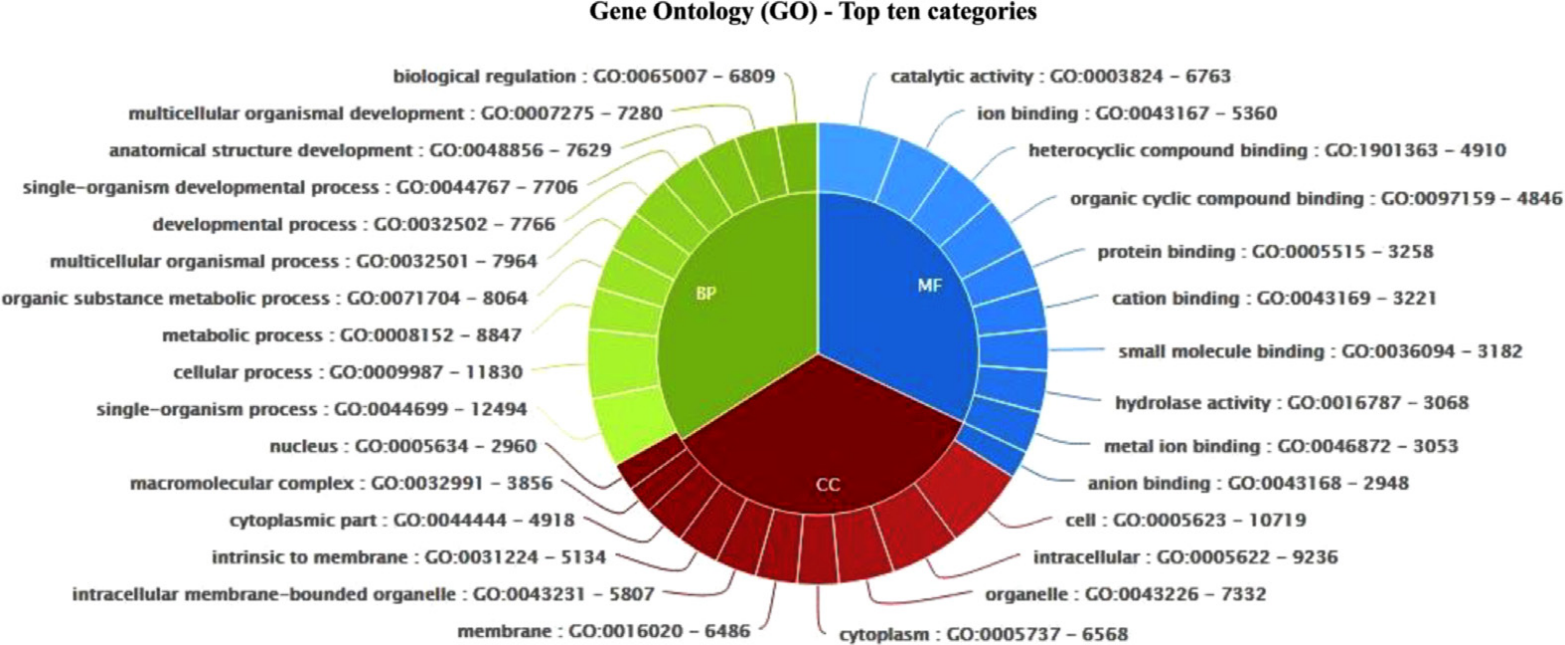
The top ten GO terms enriched under the three categories of molecular function, cellular components and biological process in the *M. incognita* proteins annotated by RefSeq.

**Fig. 3:**
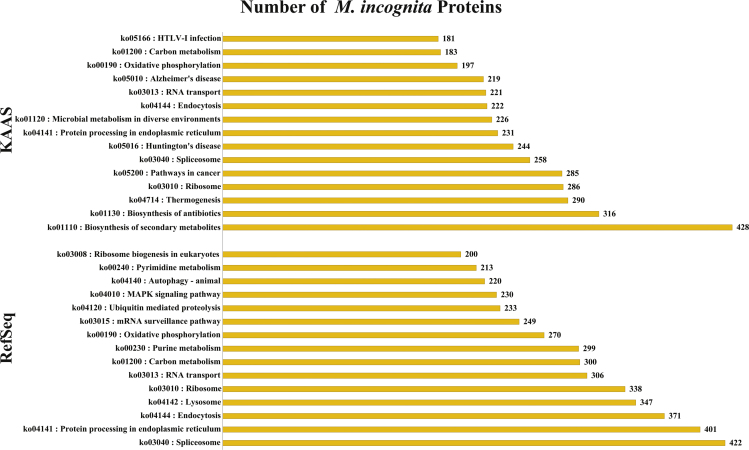
Characterization of pathways represented in the proteome data using RefSeq and KEGG Automatic Annotation Server (KAAS).

The genome of *M. incognita* was published in 2008 [1]. Later on, additional genome and transcriptome data became available for *M. incognita* [2], [3]. The annotation data *per se* is not available in the public databases, thereby limiting the direct use of sequence information by the interested researchers for making sense of their own experiment-specific transcriptome data. The latest genome analysis of *M. incognita* in 2017 [3] predicted 43,718 proteins. Using a multi-pronged strategy, we performed a comprehensive annotation of these 43,718 proteins (Supplementary information 1). A flowchart showing the summary of annotation methods, and the number of proteins that were annotated by each method is presented in Fig. 1. Using the RefSeq database of *C. elegans* and Nematoda proteins followed by NCBI-FLINK based annotation for the Gene Ontology (GO), 29,621 proteins could be annotated (Supplementary information 1, Fig. 1). GO:0003824 (catalytic activity; 6763 proteins), GO:0005623 (cell; 10,719 proteins) and GO:0044699 (biological/physiological process; 12,494 proteins) were the most enriched GO terms in the molecular function, cellular components and biological process categories, respectively. The top 10 GO terms enriched under each category are represented in Fig. 2. Characterization of pathways represented in the proteome data using RefSeq and KEGG Automatic Annotation Server (KAAS) revealed that 428 proteins mapped to the pathway ko01110 (biosynthesis of secondary metabolites), 422 proteins mapped to ko03040 (spliceosome) and 401 proteins to ko04141 (protein processing in endoplasmic reticulum) (Fig. 3).

**Fig. 4:**
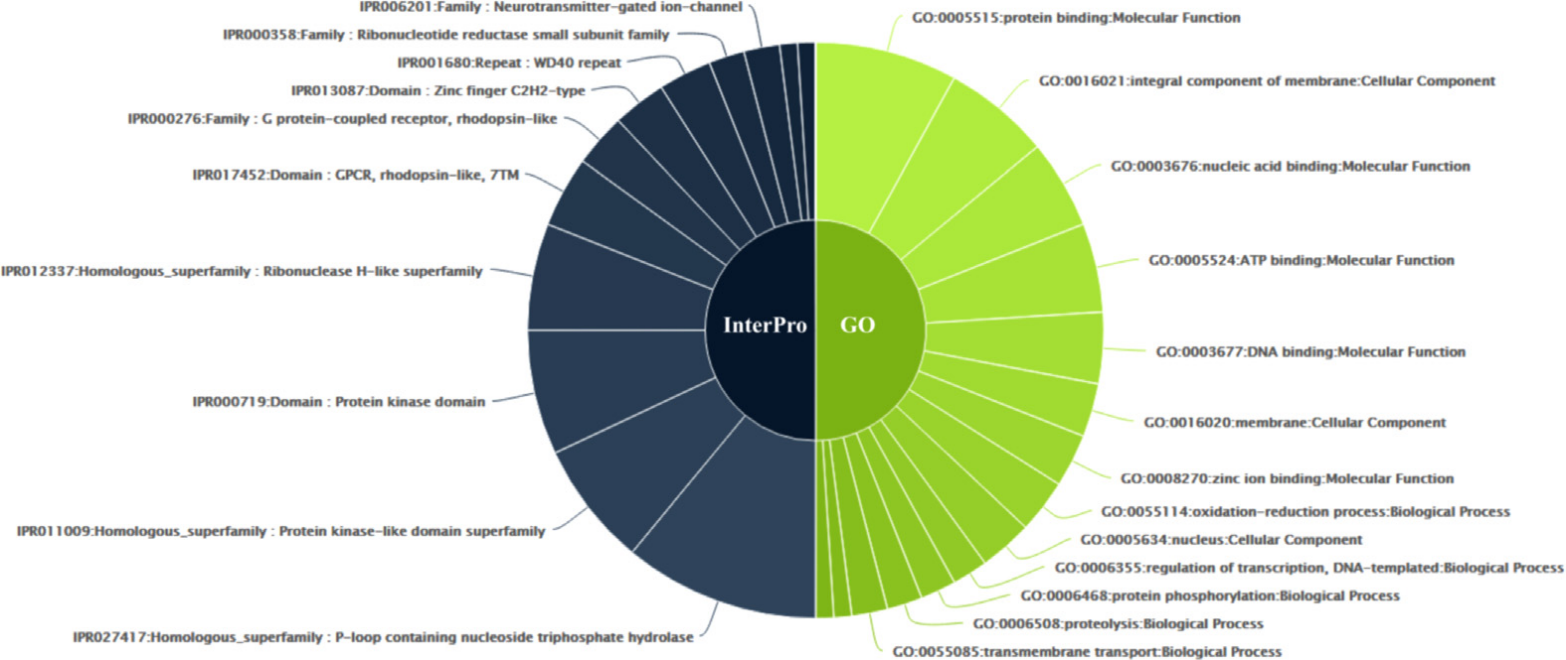
The top protein domains found in *M. incognita* proteome dataset using InterPro protein domain analysis (grey half-circle). The topmost GO terms enriched in the protein domains identified by InterPro analysis are represented by green half-circle.

In addition to RefSeq, annotation of *M. incognita* proteome data using InterProScan identified 20,163 protein domains. P-loop containing nucleoside triphosphate hydrolase superfamily (IPR027417) was the most enriched protein domain in *M. incognita*, followed by protein kinase-like (IPR011009) and protein kinase domains (IPRO00719) (Fig. 4). The analysis of GO enrichment of the protein domains by InterPro analysis showed that GO:0005515 (molecular function-protein binding), GO:0016021 (cellular component-integral component of membrane) and GO:0055114 (molecular function-oxidation-reduction process) were the three most enriched GO categories according to the enriched protein domains (Fig. 4, Supplementary information 2). The annotation of *M. incognita* proteome dataset using KAAS server identified 9119 proteins.

**Fig. 5:**
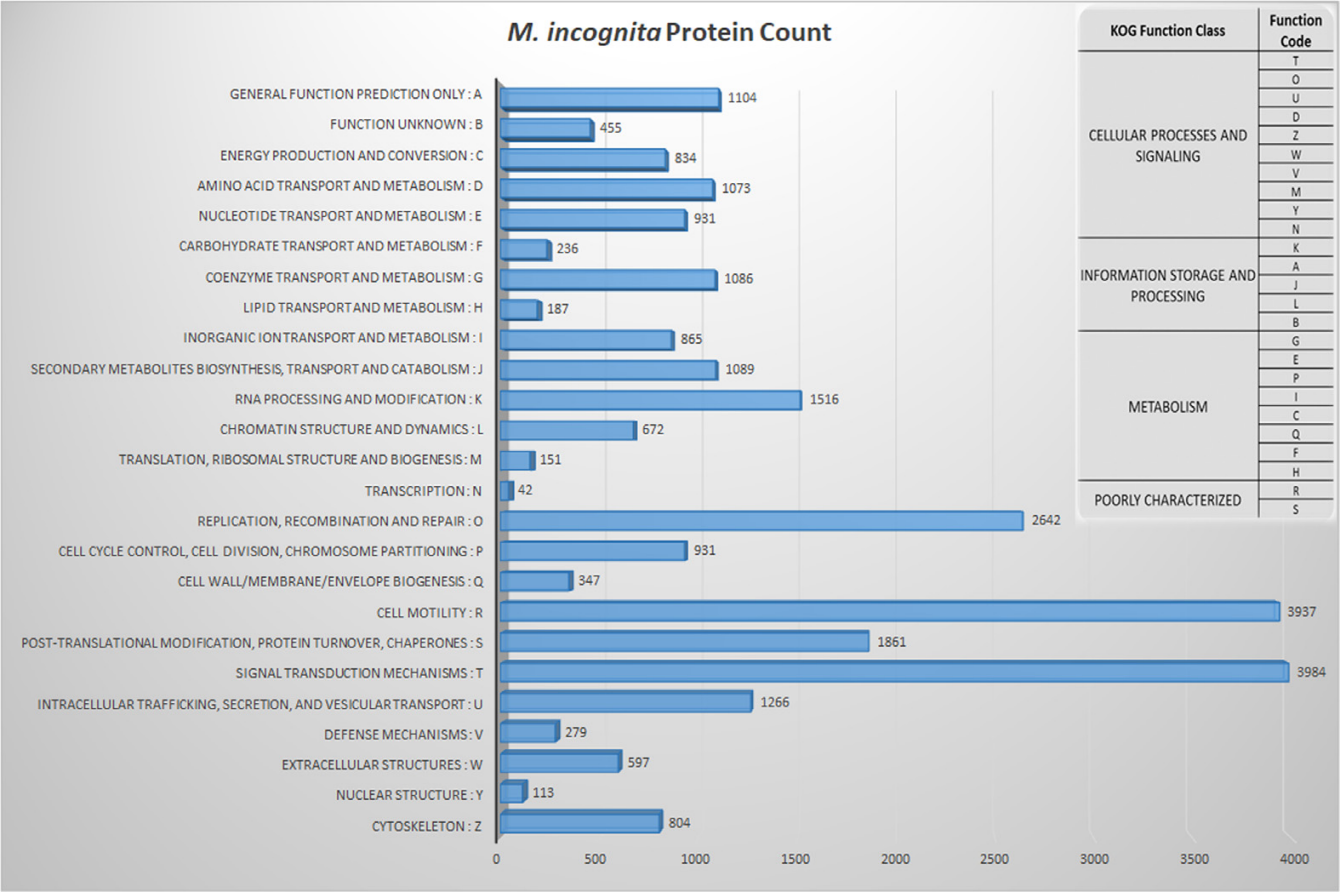
The characterization of *M. incognita* proteins into functional classes by using EuKaryotic Orthologous Groups (KOG). Bars represent proteins in each KOG function class. The box shows the KOG function class and function code.

**Fig. 6:**
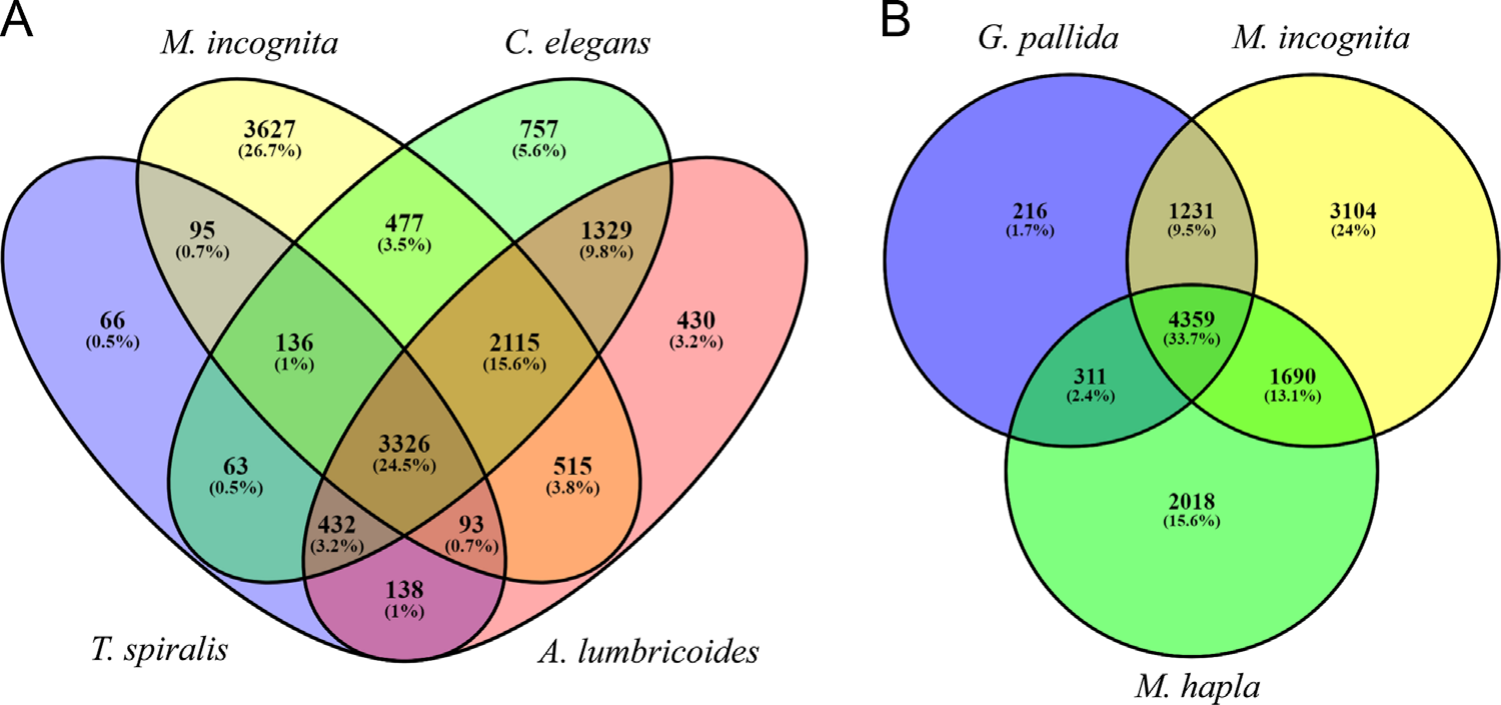
Venn diagram showing the number of ortholog groups of protein sequences conserved between *M. incognita* and other nematodes. **A.***M. incognita* (clade IV) proteome compared to *Trichinella spiralis* (clade I), *Ascaris lumbricoides* (clade III), and *Caenorhabditis elegans* (clade V), and **B.** with plant parasitic nematodes *Globodera pallida* and *M. hapla* (all clade IV). The analysis was performed by OrthoMCL.

Using EuKaryotic Orthologous Groups (KOG) to find ortholog and paralog in *M. incognita* proteome dataset annotated 30,400 proteins. These proteins were then assembled into respective KOG functional classes. The highest number of proteins (3984) grouped into signal transduction mechanisms, followed by 3937 proteins enriched in cell motility category (Fig. 5). A search for ortholog groups of protein sequences by OrthoMCL was carried out by comparing *M. incognita* proteome (clade IV) to protein sequence data of *Trichinella spiralis* (clade I), *Ascaris lumbricoides* (clade III), and *Caenorhabditis elegans* (clade V), and with plant parasitic nematodes *Globodera pallida* and *M. hapla* (both clade IV). *M. incognita* shared 3650 ortholog protein families with *T. spiralis*, 6054 with *C. elegans* and 6149 with *A. lumbricoides*, whereas 3326 ortholog protein families were common to all the compared nematodes (Fig. 6A). However, when compared to the plant-parasitic nematodes of clade IV, 4359 ortholog protein families were common between all the compared plant-parasites (Fig. 6B). Lastly, the completeness of our annotation was validated by looking for gene classes already reported in the *M. incognita* genome/transcriptome. We could find 45 RNAi effector proteins whereas 27 have been reported earlier [4]. Similarly, 458 CAZymes and 108 *M. incognita* effector proteins were identified (Supplementary information 1).

**Fig. 7:**
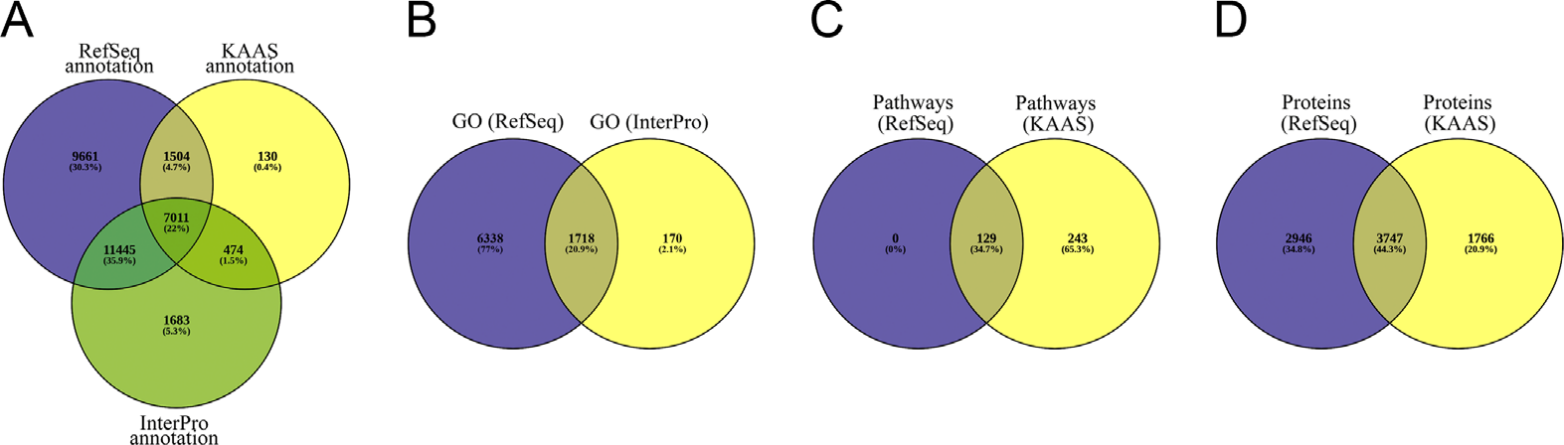
Venn diagrams showing the improvement of *M. incognita* proteome data annotation by using multiple approaches. **A.** annotation by RefSeq, KAAS and InterPro **B.** comparison of Gene Ontology (GO) terms between proteins annotated by RefSeq and InterPro approaches **C.** pathways identified by RefSeq and KAAS, and **D.** Proteins identified in the pathways represented in (C).

In summary, by using multiple approaches for proteome annotation, we have increased the number of characterized proteins in the *M. incognita* proteome dataset to 73% as compared to 67.7% by the standard RefSeq based method (Fig. 1). We characterized 2287 additional proteins and 170 gene ontologies (Fig. 7A, B) based on domain level analysis using InterProScan, and added information on additional 243 pathways (Fig. 7C), and 1766 proteins (Fig. 7D) by using KAAS.

## 2 Experimental design, materials and methods

The protein sequence file used for annotation was obtained from the INRA *Meloidogyne* Genomic Resources page (https://meloidogyne.inra.fr/Downloads/Meloidogyne-incognita-V2-2017) [3]. We initiated the annotation by blasting the INRA CDS sequences, using BLASTP, against the RefSeq database of *C. elegans* and Nematoda proteins with a cut-off set to e-value = 10^−3^ and query coverage of > 60% [5]. To obtain functional annotation and GO term, we used NCBI-FLINK [https://www.ncbi.nlm.nih.gov/Structure/flink/flink.cgi] through which the genes were assigned GO IDs for each of three ontology terms (biological process, molecular function and cellular component) and retrieved the KEGG pathways. To enrich and refine the obtained annotation further, domain level analysis was done using InterProScan [6], wherein INRA sequences were scanned for protein domains. To perform proteome annotation using secondary databases, protein sequences were annotated by similarity to characterized proteins. The KOG database (eukaryotic representatives of the COG database) [7] is one of the secondary databases, wherein orthologous gene products are classified into 25 functional categories. The INRA protein sequences were queried against KOG database for functional classification at e-value of 10^−3^. To better understand functions and interactions, all annotated genes were also mapped against the KEGG database for a pathway-based analysis using the online KEGG Automatic Annotation Server (KAAS) (http://www.genome.jp/kegg/kaas/). KEGG Orthology (KO) assignment was obtained using the GHOSTX which is a homology search tool, detects remote homologues like BLAST but 100 times more efficient than BLAST and bi-directional best hit (BBH) method [8]. The output of KEGG analysis consisted of KO assignments and KEGG pathways. Lastly, to identify ortholog protein groups among all the clades of Nematoda, we used OrthoMCL tool [9] which identifies orthologs based on blast and Markov Chain Clustering (MCL). Protein sequences of nematodes from different clades ranging from 1 to 5, including *A. lumbricoides, C. elegans, G. pallida, M. hapla,* and *T. spiralis* were downloaded from Wormbase ParaSite (http://parasite.wormbase.org/index.html). OrthoMCL was run with default parameters. The proteins belonging to RNAi pathway were analysed by comparing with *C. elegans* RNAi pathway homologues. The carbohydrate active enzymes (CAZymes) were identified by using Carbohydrate Active Enzymes database (http://www.cazy.org/) [10]. The nematode effectors were identified by first creating a local nematode effector protein database by using known plant-parasitic nematode effector proteins, and using it as a query to probe *M. incognita* proteome at e-value = 10^−3^, query coverage > 60%, and percent identity > 90%.

## Figures and Tables

**Supplementary Information 2:**
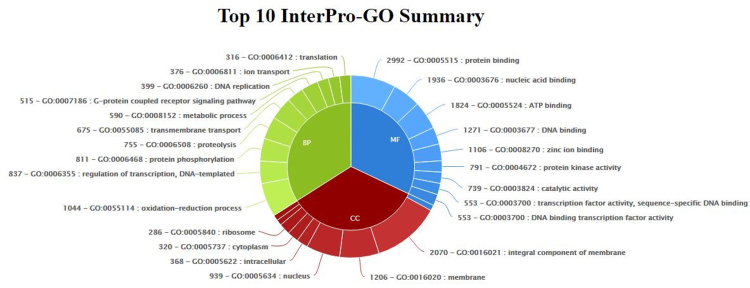
The top ten GO terms enriched under the three categories of molecular function, cellular components and biological process in the M. incognita proteome data annotated by InterPro domain and family search.
